# Global Experiences of Community Responses to COVID-19: A Systematic Literature Review

**DOI:** 10.3389/fpubh.2022.907732

**Published:** 2022-07-19

**Authors:** Yijin Wu, Quan Zhang, Meiyu Li, Qingduo Mao, Linzi Li

**Affiliations:** ^1^Center for Medical Humanities in the Developing World, School of Translation Studies, Qufu Normal University, Rizhao, China; ^2^School of International Affairs and Public Administration, Ocean University of China, Qingdao, China; ^3^Centre for Quality of Life and Public Policy, Shandong University, Qingdao, China; ^4^School of Economics and Management, China University of Petroleum (East China), Qingdao, China; ^5^Rizhao Maternal and Child Health Hospital, Rizhao, China

**Keywords:** COVID-19, community, responses, epidemic prevention, global experience

## Abstract

**Objective:**

This study aimed to conduct a systematic review of the global experiences of community responses to the COVID-19 epidemic.

**Method:**

Five electronic databases (PubMed, Embase, CINAHL, ScienceDirect, and Web of Science) were searched for peer-reviewed articles published in English, from inception to October 10, 2021. Two reviewers independently reviewed titles, abstracts, and full texts. A systematic review (with a scientific strategy for literature search and selection in the electronic databases applied to data collection) was used to investigate the experiences of community responses to the COVID-19 pandemic.

**Results:**

This review reported that community responses to COVID-19 consisted mainly of five ways. On the one hand, community-based screening and testing for Coronavirus was performed; on the other hand, the possible sources of transmission in communities were identified and cut off. In addition, communities provided medical aid for patients with mild cases of COVID-19. Moreover, social support for community residents, including material and psychosocial support, was provided to balance epidemic control and prevention and its impact on residents' lives. Last and most importantly, special care was provided to vulnerable residents during the epidemic.

**Conclusion:**

This study systematically reviewed how communities to respond to COVID-19. The findings presented some practical and useful tips for communities still overwhelmed by COVID-19 to deal with the epidemic. Also, some community-based practices reported in this review could provide valuable experiences for community responses to future epidemics.

## Introduction

The outbreak of COVID-19 was reported in December 2019 (1). Considering the fast spread of the epidemic, it was declared a public health emergency of international concern by the World Health Organization (WHO) on January 30, 2020 (2), and further announced as a pandemic on March 11, 2020 (3). Then, the confirmed cases of COVID-19 increased from 987 on January 11, 2020, to 314,181,638 on January 11, 2022, and the death toll from 17 to 5,530,773 (4). Over the past year, the number of daily new cases has shown a fluctuating trend, ranging from 300,000 to 900,000. No downward trend has been reported in sojourns, even after the COVID-19 vaccine has been administered worldwide (3). Therefore, besides promoting vaccination campaigns, the role of epidemic prevention and control actions remains important.

Community cluster infections are a significant cause of the rapid spread of the COVID-19 pandemic; they have been reported in most affected countries in the world (5). In China, the community cluster infection was the primary reason why large numbers of people were infected at the beginning of the outbreak of COVID-19. In this review, “community” refers to “residential community”. According to the definition of the WHO, community is “a group of individuals who live together in a specific geographical place, which maintains social relations among its members who recognize that they belong to such a community” (6). Thus, activities that require the community to cluster can facilitate the spread of the epidemic within the community and expose residents to the dangers of the pandemic (7, 8). Group gathering and intimate contact are widespread due to often high population density in communities, providing a high possibility for the spread of the epidemic (9). COVID-19 pandemic is highly transmissible with various transmission routes such as airborne, respiratory droplets, and direct close contact. In this sense, communities are more susceptible to the spread of the COVID-19 pandemic (10). The community spread has been reported in many countries (11).

In response to the community spread of COVID-19, the WHO launched a comprehensive guideline on community epidemic prevention, “Responding to community spread of COVID-19: Interim Guidance” on March 7, 2020 (12). Governments from many countries attached great importance to the community spread of the epidemic and took a series of strict measures to prevent and control the epidemic in communities. China, Japan, the United States, and other countries formulated plans for community epidemic prevention. Many communities around the world are exploring countermeasures to contain the epidemic. Although the epidemic is still raging around the world, some measures taken by communities have played important roles in controlling the spread of the epidemic, thus providing valuable experiences for areas still overwhelmed by COVID-19. Furthermore, global experiences of community responses to COVID-19 can also shed some light on global community responses to future pandemics.

A bulk of information exists about the community responses to COVID-19 in different countries. It is necessary to summarize the reported experiences of community responses. Several reviews explored the experiences of certain aspects of community responses to COVID-19, such as face masks (13), hand hygiene (14), contact tracing (15), and risk communication (16). However, a comprehensive review of the experiences of community responses to COVID-19 is lacking. In this study, we searched published articles about community responses to COVID-19 through electronic databases. The global experiences of community responses to COVID-19 will be approached from multiple aspects with different themes *via* a systematic review of the collected data.

## Methods

### Study Design

In this study, a systematic review was used to investigate the experiences of community responses to COVID-19 (17). Systematic review is a research method for the systematic and objective interpretation of literature content through the classification process of coding and identifying themes (18). Given its potential for interpreting the literature systematically and addressing the richness and uniqueness of the data, systematic review has been widely used in health-related disciplines and fields, including nursing (19), health promotion (20), and health services and management (21). In recent years, health researchers have begun to use this method to describe and summarize the practices and experiences in the health field (22–25).

This study was conducted based on the methodological approach pioneered by Khan et al., which consisted of the following five stages (26). Stage 1 was to identify the research question, which could guide the way of data collection. Our research question was: How did communities respond to COVID-19 worldwide? Stage 2 was to identify relevant publications. We adopted a scientific strategy to search for the relevant literature, which could answer the research question as much as possible, and employed a systematic mechanism to exclude literature not related to our research question. Stage 3 was to assess the quality of the selected literature. Stage 4 was to chart and analyze the extracted data. A narrative synthesis was used to develop themes and subthemes in this study. The last stage was to report the results and interpret the findings.

### Data Collection

#### Literature Search

Five electronic databases (PubMed, Embase, CINAHL, ScienceDirect, and Web of Science) were used to conduct a thorough search of the published literature related to COVID-19 control and prevention in global residential communities from inception to October 10, 2021. According to the objectives of this review and the PICo template for systematic reviews (27), the search strategy consisted of sets of terms for Population (actors involved in community's response to the COVID-19), the phenomenon of Interest (community measures in response to COVID-19) and the Context (residential community). Literature search terms (MeSH terms) were used to retrieve relevant studies as much as possible (see Supplementary Material 1). All collected articles were exported into reference management software NoteExpress for convenience management.

#### Literature Selection

**Figure 1:**
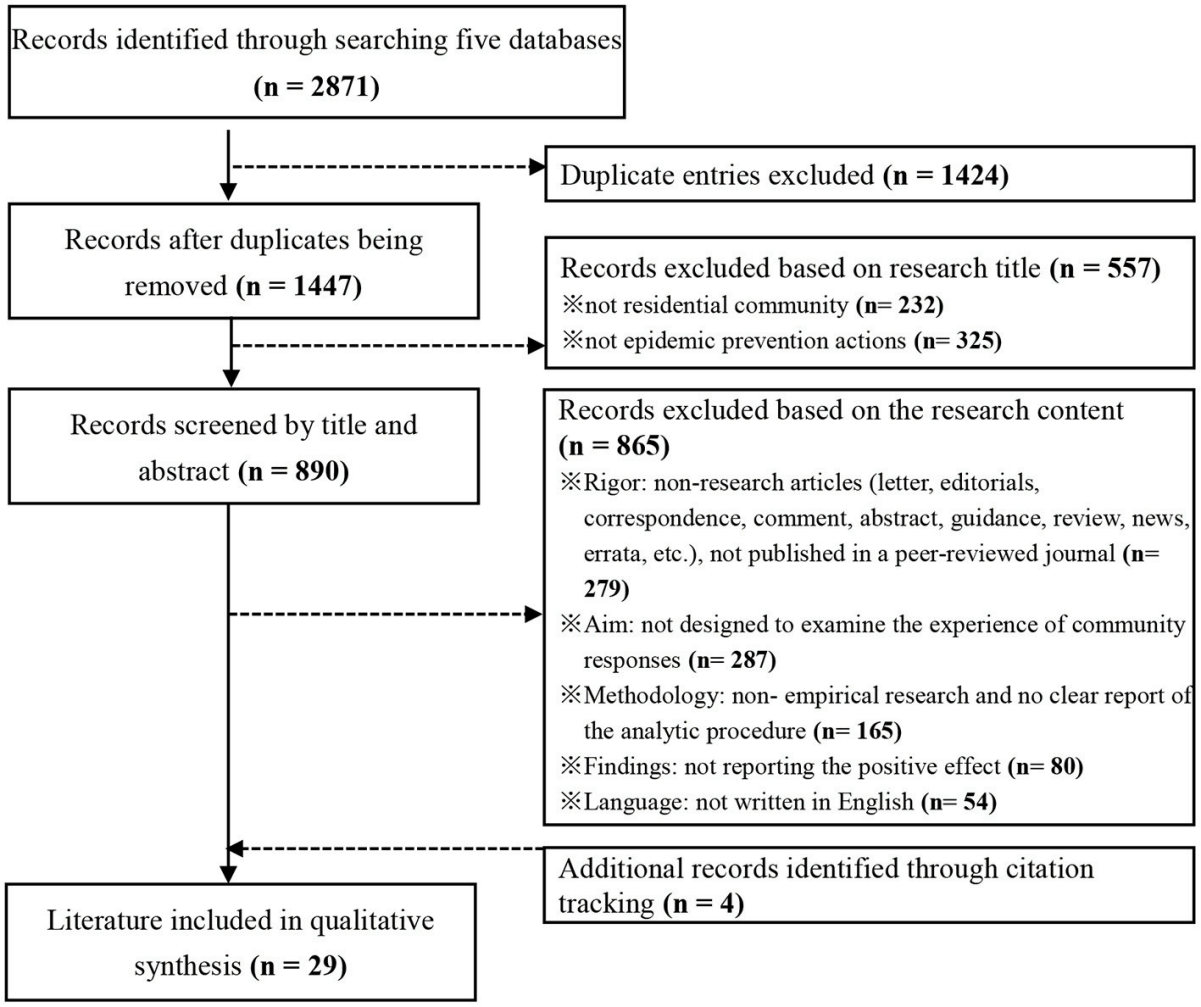
Flow chart of the literature identification and selection.

A three-step process proposed by Lockwood et al. (28) was used to review and select the relevant literature closely related to the research question (Figure 1).

First, 2,871 articles were obtained in total. The title of each article was carefully read to exclude the duplicated entries. A total of 1,424 duplicated entries were found, and thus 1,447 records were screened with their titles.

Second, an initial review of the titles was conducted by two independent reviewers to exclude the entries whose titles showed that they were clearly outside the scope of the study. If an agreement on the exclusion of an article could not be reached between those who had read the title, they included it for further review of its full text. A total of 557 articles were excluded at this stage. Among these, 232 records did not involve the residential community. The topics included other forms of the community during COVID-19, such as “oncology community” (29), “virtual community” (30), “nursing community” (31), “deaf community” (32), “East African community” (33), and others. Furthermore, 325 records had no strong correlation with epidemic prevention, and the topics included “risks and vulnerabilities” (34), “community mobility” (35), “fear effect” (36), “mental health” (37), “economic impacts” (38), “living experiences” (39), and so on.

Third, the full texts of 890 remaining articles were read by two independent reviewers to exclude the entries that could not provide a valid representation of the experience of community epidemic prevention and control. Any disagreements at any stage were resolved through discussion with a third reviewer (26). In this step, non-research articles, non-empirical research articles, articles without a clear report of the analytic procedure, and articles not reporting the positive effect of community responses were excluded. Besides, 54 non-English articles were also excluded. Then, 25 articles met the inclusion criteria for this study. In addition, four additional articles were identified through citation tracking.

### Quality Appraisal

As this review involved quantitative, qualitative, and mixed-method studies, the Mixed Methods Appraisal Tool (MMAT) was used to appraise the quality of the included literature (40). MMAT was purposely designed as a checklist for concomitantly appraising the methodological quality of different types of empirical studies (qualitative, quantitative, and mixed-method studies) included in systematic mixed-study reviews (40). The MMAT (Version 2018) provides a set of criteria for screening questions, and a score is made for each study. QZ and YW independently appraised the quality of the included studies, and appraisal disagreements were discussed until consensus was reached (Supplementary Material 2). Given the good quality score of each study, no studies were excluded (41).

### Data Extraction

Studies were extracted according to the following characteristics: authors, year, country/region, study quality (MMAT score), study design, study setting, data collection, participants, and key findings (Table 1). The first reviewer conducted data extraction, which was re-checked by a second reviewer (YW).

**Table 1:** Characteristics of included studies.

**References, country/region, study quality ^**(MMAT)**^**	**Study design**	**Setting**	**Data collection**	**Participants**	**Key findings**
Ansari et al. (42) The United Kingdom***	Quantitative descriptive	Communities in Kent County	Data mining from the number of cases collected by the Community Ophthalmology Team	Community ophthalmology patients (*n* = 6,262)	Ophthalmology services delivered by IP specialized optometrists could efficiently treat and manage the increasing number of urgent cases and deal with the reduced capacity for emergency treatment of patients
Apata et al. (43) The United States**	Mixed method	A community dialysis center in Georgia	Observations, field note, and face-to-face interviews	Community dialysis patients (*n* = 751)	Community dialysis facilities could implement measures (COVID-19 screening, universal masking, telemedicine, isolation room for dialysis of patients with suspected or confirmed COVID-19, etc.) to successfully control COVID-19 infection and serve the community dialysis patients
Aulandez et al. (44) The United States, Canada**	Qualitative	11 indigenous communities	Observations, field note, and face-to-face interviews	Patients (*n* = 23)	Holistic wellness boxes (cards with teachings on dealing with stress, lavender essential oil for practicing mindfulness, storybooks for encouraging children, resources to prevent COVID-19, etc.) could relieve the psychosocial and physical health risks of residents in indigenous communities
Bahagia et al. (45) Indonesia***	Qualitative	Urug and Cipatat Kolot villages	Observations, in-depth interviews, and documentation	Customary heads of the Urug and Cipatat Kolot people (*n* = 4) and community member	Different types of social organizations took part in food provision and allocation to the vulnerable groups (orphans, elderlies, widows, etc.)
Baratta et al. (46) Italy***	Mixed method	Community pharmacies in the Piedmont region	Questionnaire and online interviews	Community pharmacists (*n* = 286)	Protective strategies taken in the pharmacies (hygiene measures, PPTs, social distancing, etc.) could effectively halt the spread of the virus among pharmacists and ensure the pharmacies to provide pharmacy services to the community patients safely
Biro-Hannah (47) The United Kingdom ***	Qualitative	A UK community	Participatory observation	Adult mental health service users (*n* = 6)	Online group art therapy delivered by an online art therapy group mitigated the psychological effects of COVID-19 on the community adult residents
Cheng et al. (48) China****	Qualitative	Communities across 5 major cities in Zhejiang province	Semi-structured interviews	Government officials (*n* = 12), agency directors (*n* = 30), civil servants (*n* = 70), and citizens (*n* = 35)	Multiple measures involving social organizations such as temperature checks at the entrances of the communities, contact tracing, home quarantine, and safeguarding food supply played an important role in the successful prevention of the epidemic in the community
Cheng et al. (49) Hong Kong**	Quantitative non-randomized	Communities in Hong Kong	Data mining from multiple open data	Hong Kong citizens (*n* = 7.45 million)	Community-wide mask wearing contributed to the control of COVID-19 by reducing the amount of emission of infected saliva and respiratory droplets from individuals with subclinical or mild COVID-19
Durmuş et al. (50) Turkey****	Quantitative non-randomized	Communities across Turkey	Data mining from Google Mobility Reports	Android devices users in Turkey	Community-based social distancing significantly decreased the effective reproduction number (Rt) of COVID-19 by reducing human mobility, and thereby prevented many people from becoming infected
Frimpong et al. (51) Sierra Leone****	Qualitative	Two communities called Cockle Bay and Portee	In-depth interviews and focus group discussions	Community-based organization leaders (*n* = 20), experts (*n* = 4), and government officials (*n* = 4)	Multiple measures lead by community-based organizations, such as providing epidemic prevention knowledge, mobilizing COVID-19 response actions, supplying sanitary items, risk communication, and supporting vulnerable residents, played a major role in responding to COVID-19
George et al. (52) The United States*****	Quantitative	Communities across 27 states	Data mining from Columbus Electronic Health Record	Community rheumatology patients (*n* = 60,002)	Telehealth care offset the large disruptions in care during the COVID-19 pandemic to some extent and protected the community patients
Ha et al. (53) Vietnam*****	Qualitative	Communities in Que Vo and Phuc Son districts	Semi-structured interviews	Residents, community representatives, health authorities, etc. (*n* = 36)	Community prevention measures, such as early detection, isolation, quarantine, and risk communication, played an important role in the prevention and control of COVID-19
Hutchings et al. (54) Australia****	Qualitative	Communities served by SLHD in New South Wales	Observations and field note	Community patients with COVID-19 (*n* = 162)	Community-based virtual health care, including telemedicine in combination with remote patient monitoring, was a feasible and safe approach for managing less severe cases of COVID-19
Juhn et al. (55) The United States***	Quantitative descriptive	Communities in Southeast Minnesota	Questionnaire survey	Community residents aged more than 50 years (*n* = 2,325)	Community based social distancing, mask wearing, and hand hygiene might significantly mitigate the risk of COVID-19
Kwok et al. (56) Hong Kong***	Quantitative non-randomized	Hong Kong communities	Questionnaire survey	Community residents (*n* = 1,715)	Community-based measures, such as personal hygiene, travel avoidance, and social distancing, might slow the transmission of COVID-19
Lim et al. (57) The United Kingdom*****	Qualitative	Community pharmacies in South East of England	Semi-structured telephonic interviews	Community pharmacy team members (*n* = 14)	Innovative services (home medication delivery services, tailored services, and telephone and video-consultations) provided by community pharmacies could support the community dementia patients safely during the epidemic
McCalman et al. (58) Australia****	Qualitative	An indigenous community Yarrabah, Far North Queensland	Semi-structured telephonic interviews	Primary healthcare staff (*n* = 13), community and government leaders, and community members (*n* = 5)	Multiple measures such as community lockdown, COVID-19 testing, quarantine, risk communication, providing epidemic prevention knowledge, and supporting residents' wellbeing (food supply, mental health services, etc.) prevented Yarrabah community from having a single confirmed case
McConachie et al. (59) The United States**	Mixed method	A community hospital	Filed work and documentation from Beaumont community hospital	Clinical specialists, pharmacists, and community patients	Beaumont community hospital restructured its work workflow, shifted its medication supply, and innovated pharmacokinetic dosing services to provide effective pharmaceutical services for its residents during the pandemic
Narasri et al. (60) Thailand****	Qualitative	Communities with food insecurity challenges in Bangkok	Focus group interviews	Community volunteers (*n* = 12), community leaders (*n* = 4), and health providers (*n* = 4)	Multiple measures such as community pantry of sharing, community gardening, and collaboration within external organizations successfully achieved sustainable food security of the community
Omboni et al. (61) Italy***	Quantitative non-randomized	Communities served by a telehealth platform	Data mining from the TEMPLAR Project database	Community patients using telehealth service	Telehealth performed in community pharmacies was a feasible and useful solution for monitoring the health status (ambulatory blood pressure, spirometry, sleep oximetry, etc.) of community patients during the epidemic
Patel et al. (62) The United States***	Quantitative descriptive	A large community pharmacy in Arizona	Questionnaire survey	Community residents (*n* = 622)	Pharmacy-provided point-of-care testing services for COVID-19 expanded patient access to testing during the pandemic in a lower-income community
Peng et al. (63) China****	Quantitative non-randomized	Communities across China	Data mining from Tencent and Ifeng real-time tracking and National Health Commission data	Community residents	Intensive community screening was extremely effective in suppressing the spread of COVID-19 virus
Pruitt et al. (64) China***	Quantitative descriptive	Communities across Montana	Web-based questionnaire survey	Professionals providing suicide prevention services to American Indians (*n* = 80)	Most telehealth users reported that telehealth was effective in preventing suicides among American Indian communities in Montana during COVID-19. Telehealth providers perceived suicide prevention services through telehealth as effective as face-to-face care
Vanhamel et al. (65) Belgium****	Qualitative	Orthodox Jewish communities of Antwerp	In-depth interviews, key informant interviews, and community mapping	Community leaders (*n* = 7) and community members (*n* = 9)	Risk communication performed by community leaders proved to be of great importance to foster a feeling of trust in the government's response measures and facilitate the coverage and uptake of pandemic control measures
Villani et al. (66) Ireland***	Qualitative	Traveler and Roma Communities	Observation notes, NGOs' briefings, and minutes of meetings	Government officials, community health staffs, and NGO members	In Traveler and Roma communities, public health measures (equipment of waste collection, running water, and mobile isolation units), economic and social support, culturally appropriate communications, and lobbying for prevention measures) contributed to minimizing the health inequities during the pandemic
Wallis et al. (67) The United Kingdom***	Quantitative descriptive	Communities in London	Questionnaire survey	Community patients who were tested by NHS trusts (*n* = 2,053)	Community testing allowed widespread testing for COVID-19 while minimizing patient transport, hospital admissions, and staff exposures. Thus, it was an important and feasible approach to mitigate the epidemic
Wong et al. (68) Hong Kong***	Mixed method	A community isolation facility and a community treatment facility	Observations, field note, and data mining from the CIF and CTF	Community patients of the CIF and CTF	Community isolation and treatment facilities accompanied by meticulous infection control measures (staff training, audit, staff and patient hand hygiene, and direct observation of donning and doffing) was a feasible and safe approach to combat the epidemic
Zhang et al. (69) China***	Qualitative	Haiyu community in Shenzhen city	Observations and field note	General practitioner, community manager, and government officials	Community containment strategies, including temperature checking, mask wearing, contact tracking, quarantine, and isolation treatment, limited community transmission of the virus
Zhu et al. (70) China***	Quantitative non-randomized	Communities in Anhui province	Data mining from the data of two trauma centers and National Health Commission	Patients in two trauma centers and confirmed cases	Community quarantine strategy was effectively implemented and significantly slowed the outbreak of COVID-19 in Anhui province. However, the implementation and maintenance of the strategy was costly

The symbols *, **, ***, ****, and ***** refer to scores of 20, 40, 60, 80, and 100%, respectively, obtained on the Mixed Methods Appraisal Tool (MMAT), version 2018; the study design was categorized according to this criterion presented in the MMAT, version 2018.

### Data Synthesis

As the included studies were a mixture of quantitative, qualitative, and mixed-method studies, a narrative synthesis of the data was conducted using a thematic approach (71). The narrative synthesis aimed to present a descriptive summary of findings across the included studies and generate themes relevant to the aims of this review (72).

In this review, we followed the method for qualitative data synthesis proposed by Elo and Kyngäs, which consisted of three phases (73). In the preparation phase, the core tasks were “deciding on what to analyze in what detail” and “selecting the unit of analysis”; we focused our analysis on the actions/measures against the COVID-19 pandemic (74). In the organizing phase, the core tasks were open coding, creating categories, and the abstraction. For open coding, two researchers read through the article and wrote down headings about community epidemic prevention and control independently (74). For category creation, the lists of headings were grouped under higher-order subthemes, which could provide a good knowledge of community responses to COVID-19 pandemic (75). For data abstraction, sub-themes with content similarities were grouped as main themes (76). In the reporting phase, the analytical process and categories of the results were reported in detail.

To ensure methodological rigor, two researchers (QZ and YW) independently coded all the included literature. Coding disagreements were discussed until consensus was reached (77). In addition, codes were expanded and changed to ensure that they were extremely exhaustive during the coding process (78). PRISMA checklist was provided in Supplementary Material 3 (79).

## Results

The characteristics and quality assessments of 29 included studies are shown in Table 1. All of them were published after 2019. Six studies were conducted in the United States, five in China, four in the United Kingdom, three in Hong Kong, two in Italy and Australia, respectively, and one in Belgium, Ireland, Thailand, Turkey, Vietnam, Indonesia, and Sierra Leone, respectively. Thirteen of the 29 included studies used qualitative study design, twelve employed quantitative study design, and four used mixed methods. MMAT findings showed that the quality of included studies was variable, with a mixture of studies having 100% (*n* = 3), 80% (*n* = 8), 60% (*n* = 14), and 40% (*n* = 4) quality.

The results showed that global experience of community responses to COVID-19 epidemic were composed of the following five themes (Table 2).

**Table 2:** Synthesized themes from included studies.

**Themes**	**Subthemes**	**Theme descriptions**
Community screening of COVID-19	Biotechnology-based detection methods	Biotechnology testing services provided by community pharmacy
		Biotechnology testing services provided by National Health Service trusts
		Biotechnology testing services provided by community clinic
	Non-biotechnology-based detection methods	Community screening based on travel history and close contact information
		Community screening based on temperature and symptoms checks
Cutting off the transmission chain of the virus	Preventing the invasion of the virus into the community	Limited community closure
		Complete community closure
	Preventing cross-infection in the community	Community-based mask wearing
		Community-based social distancing
		Community-based quarantine
		Personal hygiene measures
Providing medical aid for mild cases		Community treatment facilities
		Community virtual health care
Social support for the residents	Material support	Food supply and food security
		Alcohol and drugs supply
	Psychosocial support	Risk communication
		Holistic wellness boxes, with various items to support mental wellbeing
		Online group art therapy
Protecting vulnerable residents during the epidemic	Physical health service for physically disadvantaged residents	Offline community dialysis service
		Offline community pharmacy services
		Online health status monitoring
	Mental health service for spiritually vulnerable residents	Online consulting for suicide prevention to the residents at suicide risk
		Mental health services to the residents experiencing grief and trauma

### Community Screening for COVID-19

Six studies addressed the theme of community screening for COVID-19 (48, 53, 58, 62, 63, 67). There were two subthemes relating to this theme: biotechnology-based detection methods and non-biotechnology-based detection methods.

#### Biotechnology-Based Detection Methods

Biotechnology-based methods for COVID-19 testing have been widely used in communities of developed countries, where medical resources are relatively abundant. Compared with centralized COVID-19 testing, community-based testing has its unique advantages. In a large lower-income community in Arizona, a community pharmacy provided point-of-care testing services for its residents during the COVID-19 pandemic. Pharmacists were trained to administer COVID-19 tests to their community residents. Residents received a pharmacist-guided nasal self-swab collection and got their results within 24 h by a follow-up phone call (62). The community testing program provided by National Health Service Trust in London also showed that community testing could provide an accurate picture of how many confirmed cases would exist in a community (67). In an Australian indigenous community *Yarrabah*, community-based testing was provided by a primary healthcare center. Community-based testing, aside from other measures, successfully prevented the invasion of COVID-19 into *Yarrabah* community (58). It was also suggested that the nationwide intensive community screening in China was extremely effective in controlling the spread of the COVID-19 epidemic (63).

#### Non-biotechnology-based Detection Methods

Biotechnology-based detection methods require a large amount of human and medical resources, which pose great challenges for communities. Thus, besides biotechnology-based detection methods, non-biotechnology-based detection methods were also used in community responses to COVID-19. In Vietnam, the community volunteers collected epidemic-related data from residents, including daily temperature and symptoms of household members (53). In addition, community officials collected residents' travel history and their close contact information in order to identify at-risk cases (53). The suspected cases were reported to the local health authority, which decided whether they needed testing, isolation, quarantine, or hospitalization. This ensured the early detection of high-risk cases and prevented the spread of COVID-19 in communities (53). In China, community officials aside from volunteers contacted each resident to determine whether they had been in close contact with confirmed patients. The screening information was shared with the local Center for Disease Control and Prevention (CDC) for further measures (48).

### Cutting Off the Transmission Chain of the COVID-19

This theme examined how residential communities cut off the transmission chain of COVID-19. This theme involved nine studies (48–50, 53, 55, 56, 58, 69, 70). Two subthemes underpinned this theme, that is, preventing the invasion of the COVID-19 into the community and preventing cross-infection in the community.

#### Preventing the Invasion of the COVID-19 Into the Community

Community closure was carried out to prevent the invasion of COVID-19 into the community. Different countries adopted different strategies for community closure with different stringencies. A community called *Yarrabah* in Australia implemented limited “community lockdown” measure, which required that all residents should undergo a 14-day isolation before entry or re-entry into *Yarrabah*. Note that residents who were in urgent circumstances were exempt from travel restrictions. For example, community residents suffering from illnesses were allowed to travel to the designated hospitals outside the community for short-term medical treatment (58). Some communities in China adopted complete community closure. For instance, an 18-day community lockdown was implemented in the *Yuanqiao* community in Zhejiang province of China, and all residents were not allowed to leave their homes unless for a permitted reason. The local government and community-based organizations provided food (pork, vegetables, eggs, rice, cooking oil, etc.) for the residents living in the *Yuanqiao* community to help them get through the quarantine period (48). Community closure proved effective in preventing the community spread of the COVID-19.

#### Preventing Cross-Infection in the Community

Multiple measures have been adopted to prevent cross-infection in the community. Mask wearing, an initiative from the WHO, is the most widely used measure across the global communities. More evidence has shown that community-wide mask wearing can help stop the spread of COVID-19. Data from Hong Kong communities showed that community-wide mask wearing contributed to the control of COVID-19 by reducing the amount of emission of infected saliva and respiratory droplets from infected patients (49). The surveys on communities in the United States and China also showed that community-based mask wearing limited the community transmission of the virus (55, 69).

Social distancing is another effective way for community epidemic prevention, preventing disease transmission by reducing close contact between people. Social distancing consists of a range of concrete measures, including public place closure, gathering cancellation, avoiding close contact with other people, and staying at home. Data from Google Mobility Reports in Turkey showed that the community-based social distancing interventions (e.g., public place closure. travel restrictions, and cancellation of religious activities) significantly decreased the transmission of COVID-19 by reducing human mobility, and thereby prevented residents from becoming infected (50). In addition, community-based social distancing (e.g., avoiding taking public transport and engaging in social activities, and keeping a distance of 6 feet from others in a public space) played an important role in stopping the transmission of COVID-19 in Southeast Minnesota, the United States, and Hong Kong, China (55, 56).

Quarantine, one of the oldest and most effective tools for controlling the spread of communicable diseases, has also been used to stop the community spread of COVID-19. The WHO recommends that the contacts of patients with COVID-19 must be quarantined for 14 days from their last contact with the patient. These measures are adopted by many countries, but the range of residents required to be quarantined varies from country to country. In some residential areas of developed countries such as Australia, suspected cases need to be quarantined based on biotechnology-based testing (58). However, in some developing countries, suspected patients were quarantined at home based on mass non-biotechnology-based community screening. For example, in Vietnam, residents who traveled to the pandemic epicenter needed to be quarantined at home (53). In China, close contacts, travelers from the pandemic epicenter, and residents with a high fever for a week had to undergo 14 days of home quarantine (69). It was suggested that community quarantine of residents with suspected symptoms and clear exposure history significantly reduced the risk of the outbreak of COVID-19 in Anhui province of China (70).

### Providing Medical Aid for Mild Cases

This theme concerned how residential communities provided home-based medical aid for infected patients with mild symptoms. At the beginning of the epidemic, medical facilities did not have sufficient capacity for admitting patients. Thus, some communities tried to provide medical aid for their residents with mild symptoms. Hong Kong built community isolation facilities (CIF) and community treatment facilities (CTF) for symptomatic patients who did not require advanced medical resources. CIF and CTF accompanied by meticulous infection control measures (staff training, audit, staff and patient hand hygiene, and direct observation of donning and doffing) proved to be a feasible and safe approach to combat the epidemic (68).

In addition to centralized surveillance and care, some countries explored virtual observation and care to meet the challenge of housing and personnel storage. In New South Wales, Australia, virtual health care was established by Sydney Local Health District (SLHD) to monitor the residents' body condition, including respiratory rate, oxygen saturation, pulse rate, and temperature. Patients who were quarantined at home were contacted by video conference software twice every 24 h to recognize the signs and symptoms related to their deteriorating conditions. The deteriorating patients were transferred to the local emergency department by ambulances (54).

### Social Support for the Residents

Seven studies highlighted community's social support for the residents (44, 45, 47, 48, 60, 65, 66). This theme consisted of two sub-themes, that is, material support and psychosocial support.

#### Material Support

In response to the movement restrictions imposed by the lockdown measures, some communities provided food supplies to their residents. For example, in a community called *Yuanqiao* in the Zhejiang province of China, a 18-day community lockdown was implemented. The local government and community-based organizations delivered food (e.g., pork, vegetables, eggs, rice, and cooking oil) to the residents who were not allowed to leave their homes during the quarantine period. Due to these efforts, the degree of complaints about the long-time lockdown was significantly reduced (48). In terms of food insecurity, the communities in Bangkok and Thailand adopted multiple measures (e.g., community pantry and community gardening) to ensure food security (60). In two Indonesian villages (Urug and Cipatat Kolot), the residents experienced a severe food shortage because the food markets were suspended from operations. The food shortages were addressed by charitable donations. Different types of social organizations took part in food provision and allocation to the vulnerable groups (orphans, elderly people, widows, etc.) (45).

#### Psychosocial Support

Some communities adopted “risk communication” to establish trusted communication channels with their residents. In communities of Ireland, culturally appropriate communication strategies were used to increase community trust (66). To be specific, the culture (norms, beliefs, and values) of the target population was fully considered. In this sense, communication strategy would be more likely to be accepted by the target population (66). In the Orthodox Jewish communities of Antwerp, Belgium, a community-based communication strategy was used to promote risk communication with their residents. In addition, community volunteers operated a telephone “hotline,” to which community residents could turn for help when they were in trouble. Furthermore, the risk communication performed by community leaders in Orthodox was also proved to be of great importance in fostering a feeling of resident trust in the government (65).

Along with risk communication, mental health services were provided to community residents during the epidemic. The Johns Hopkins Center for American Indian Health delivered holistic wellness boxes (e.g., cards with teachings on dealing with stress, lavender essential oil for practicing mindfulness, and storybooks for encouraging children) to indigenous communities in the United States and Canada, which relieved their psychosocial problems effectively (44). An online art therapy group from the United Kingdom delivered online therapy to the community residents. They were encouraged to share artworks they made and discuss their images and techniques used, which helped them build resilience, increase self-awareness, and improve self-esteem (47).

### Protecting Vulnerable Residents During the Epidemic

This theme addressed eight studies that focused on protecting vulnerable residents during the epidemic (43, 46, 52, 57–59, 61, 64). Two subthemes underpinned this theme: physical health service for physically disadvantaged residents and mental health service for spiritually vulnerable residents.

#### Physical Health Service for Physically Disadvantaged Residents

Residents with severe or chronic diseases are more vulnerable to the COVID-19 epidemic. Many medical institutions tried to reform their working models to sustain their medical services for vulnerable individuals. A community dialysis center in Georgia, the United States, implemented a range of measures (e.g., screening tests, wearing masks for all individuals, reducing patient wait times, telemedicine, and isolation room for suspected or confirmed patients) for infection prevention and control and could thus be able to continue providing patients with hemodialysis services (43). Also, pharmacies in *Piedmont* communities of Italy adopted some effective strategies (e.g., personal hygiene measures, disinfection in public spaces, social distancing, universal masking, and confirmed staff being placed under quarantine) to stop the spread of the virus among pharmacists, which could ensure that they could provide safe health care to their community residents (46). In addition, many community health facilities offered telehealth services for their residents during this epidemic. Community pharmacies in the South East of England reformed their service modes and implemented innovative services, which consisted of home medication delivery services, tailored services, and telephone consultations (57). This benefited community-based dementia patients a lot (57). In Italy, telehealth performed by community pharmacies was proved to a feasible and useful way to monitor the health status (e.g., ambulatory blood pressure, spirometry, and sleep oximetry) of community patients during the epidemic (61).

#### Mental Health Service for Spiritually Vulnerable Residents

Some residents felt stressed, worried, or anxious during the epidemic. Thus, communities tried to provide psychological comforts for their residents. For example, American Indians and Alaska Natives were at a higher risk of suicide than any other racial or ethnic groups in the United States (64). Thus, these individuals were provided with online consulting services *via* video conferencing platforms, telephone, e-mail, or texting, which were effective in preventing suicides (64). In addition, a social and emotional wellbeing team was established in a community called *Yarrabah* in Australia, which provided home-based mental health services for community residents experiencing elevated fear, anxiety, grief, and trauma (58).

## Discussion

### Principal Findings

This study revealed some common experiences of community responses to the COVID-19 epidemic. First, this review found that the viral screening test was mostly used to stop the spread of the virus in the community, and untimely or delayed screening may cause community spread. In addition, immediate measures were taken to cut off the transmission route of the virus, which is in line with many previous studies (80, 81). Some measures were community-oriented measures, such as community closure and home quarantine. Whole-of-society measures including mask wearing and social distancing were also implemented in the community setting. Second, it is of great importance to balance epidemic management and residents' daily lives. As mentioned earlier, some measures, such as community closure and social activity restrictions, may influence the normal lives of residents. Thus, some community services, including material and psychological support, were used to balance epidemic management and residents' lives. Third, vulnerable individuals have been given more attention during the epidemic (82, 83). Vulnerable individuals may suffer more from COVID-19, thus a range of measures have been taken, including physical health service for physically disadvantaged residents and mental health service for spiritually vulnerable residents. Fourth, the participation of multiple actors is the key to the success of community epidemic control and prevention. Many actors are involved in community response to the epidemic, including the government, community organizations, community hospitals and medical centers, pharmacists, community workers, volunteers, residents, NGOs, business organizations, and so on. The participation of multiple actors, also known as community engagement, can give full play to the strengths of different actors in different tasks of epidemic prevention, such as plan designing, trust building, risk communication, surveillance and tracing, resource supply, trust building, and protection of vulnerable groups (84–87).

The global experiences of community responses to COVID-19 is a rich database for future epidemic prevention and control in the community. However, no “one-size-fits-all” model exists for community epidemic prevention and control, and many of these experiences cannot be replicated directly in every instance. Some preconditions need to be considered in the design of community measures.

First, measures taken by communities need to be commensurate with the affordability of the national economy. Some measures such as social distancing and societal closure have proven highly effective at reducing the community transmission of COVID-19. However, these measures may cause social and economic damage (88, 89). For example, a study showed that the lockdown measure in Chile was associated with a 10–15% drop in local economic activity, which was twice the reduction in local economic activity suffered by municipalities that were not under lockdown. A 3-to-4 month lockdown had a similar effect on economic activity than a year of the 2009 great recession (90). In low-and middle-income countries, strict social distancing measures (e.g., nationwide lockdown) in response to the COVID-19 pandemic are unsustainable in the long term due to knock-on socioeconomic and psychological effects. Compared with prolonged lockdown, zonal or local lockdowns may be a better option for these countries (91). Therefore, resources and economic capacity should be taken into full account when designing and taking epidemic prevention measures.

Second, existing health system capabilities, as the basis of prevention measures, cannot be ignored. For example, a strategy of limited lockdown of an objectively identified selected high-risk population might be a cost-effective option compared with a generalized lockdown. However, limited lockdown is more commonly used in developed countries rather than in developing ones. It can involve comprehensive biotechnology-based screening for suspected cases and thorough tracing of contacts based on adequate medical resources and a well-supported, community-based team of trained personnel, which are often not available in developing countries (92, 93). Health system capabilities define possible boundaries for community prevention measures.

Third, a country's social culture needs to be considered. For example, epidemic-relevant information registration is accepted by East Asian countries such as China, Japan, and Singapore (94, 95) but may be considered an infringement of privacy in Western countries (96, 97). In addition, home quarantine, which is widely used in China, India, and Vietnam, may be considered a restriction on personal freedom in Western countries (98, 99).

Fourth, community epidemic prevention and control need to be commensurate with the national concepts of epidemic prevention. Taking “cutting off the transmission route of the virus” as an example, community closure, social distancing (public places closure and gathering cancellation), quarantine, and travel restrictions are collectively referred to as community containment strategies (100). However, personal protective measures (handwashing, cough etiquette, and face mask), social distancing (maintaining physical distance between persons in community settings and staying at home), and cleaning and disinfection in community settings are collectively referred to as community mitigation strategies (9). Some countries (e.g., China and Singapore) tend to use community containment strategies, whereas others (e.g., the UK) prefer to use community mitigation strategies (101).

Finally, the unique circumstances of each community should be considered. Just as CDC Community Mitigation Framework (the United States) reported, each community is unique, and prevention strategies should be carried out based on the characteristics of each community (102). Specifically, different communities vary significantly in economic conditions (103), population density (104), vulnerability degree (105), physical environment (106), health facilities (107), and so on. Thus, these factors should be fully considered by policymakers when making prevention measures. In this sense, it seems that no “one-size-fits all” approach exists to fight COVID-19 in the community, and multiple contextual factors should be taken into full account.

### Strengths and Limitations

In this review, we made a comprehensive analysis of community responses to COVID-19, which could provide not only a comprehensive understanding of community responses to COVID-19, but also insightful findings that could not be obtained by a review focusing mainly on a single measure. Although this review followed a rigorous process of systemic literature review, it could not be guaranteed that this review could fully reflect “global experiences of community responses to COVID-19.” As with all literature reviews, five electronic databases rather than all electronic databases were used for searching the literature due to the huge cost of searching all the databases (108, 109). Accordingly, some relevant studies might be missing in this review. The eligibility criteria were used for literature selection. Therefore, some relevant studies (gray literature, non-English literature, etc.) could not be included in this review (110, 111).

## Conclusions

Communities around the world took multiple measures to fight against the epidemic. Community responses to COVID-19 consisted mainly of five ways. On the one hand, community-based screening and testing for Coronavirus was performed; on the other hand, the possible sources of transmission in communities were identified and cut off. In addition, communities provided medical aid for patients with mild cases of COVID-19. Moreover, social support for community residents, including material and psychosocial support, was provided to balance epidemic control and prevention and its impact on residents' lives. Last and most importantly, special care was provided to vulnerable residents during the epidemic. The findings presented some practical and useful tips for communities still overwhelmed by COVID-19 to deal with the epidemic. Also, some community-based practices reported in this review could provide valuable experiences for community responses to future epidemics.

## Data Availability Statement

The original contributions presented in the study are included in the article/Supplementary Material, further inquiries can be directed to the corresponding author.

## Author Contributions

QZ conceived the study and all authors participated in its design. YW drafted the manuscript. ML, QM, and LL have been involved in discussing earlier versions of the text. All authors read and approved the final manuscript.

## Funding

This research was supported by the Natural Science Foundation of Shandong Province, China (Grant ID: ZR2021QG015). The funding body did not influence this paper in any way prior to circulation.

## Conflict of Interest

The authors declare that the research was conducted in the absence of any commercial or financial relationships that could be construed as a potential conflict of interest.

## Publisher's Note

All claims expressed in this article are solely those of the authors and do not necessarily represent those of their affiliated organizations, or those of the publisher, the editors and the reviewers. Any product that may be evaluated in this article, or claim that may be made by its manufacturer, is not guaranteed or endorsed by the publisher.

## Abbreviations

COVID-19: corona virus disease 2019
PHEIC: public health emergency of international concern
WHO: World Health Organization
MMAT: Mixed Methods Appraisal Tool
UK: United Kingdom
PPE: personal protective equipment
Rt: effective reproduction number
NGOs: non-government organizations
NHS: National Health Service
CIF: community isolation facility
CTF: community treatment facility
CDC: center for disease control and prevention
VHC: virtual health care
CAIH: center for American Indian health
LMICs: low and middle-income countries
CMH: Community mental health.
