# Supplementary material for: Global Experiences of Community Responses to COVID-19: A Systematic Literature Review
Source: Front Public Health. 2022 Jul 19;10:907732. doi: 10.3389/fpubh.2022.907732 (PMC9343721; doi:10.3389/fpubh.2022.907732)
Supplement: Supplementary file 1 [file Data_Sheet_1.docx]

**Supplementary Material 1: Search terms used for search strategy across all databases**

The search terms used were “COVID-19” OR “2019-nCoV” OR “coronavirus” OR “Delta” AND “community” AND “action” OR “actions” OR “response” OR “responses” OR “respond” OR “responding” OR “strategy” OR “strategies” OR “measure” OR “measures” OR “countermeasures” OR “approach” OR “practice” OR “practices” OR “experience” OR “experiences” OR “lessons” OR “learning” OR “contribution” OR “role” OR “use” OR “prevent” OR “prevention” OR “preventing” OR “control” OR “contain” OR “containment” OR “mitigate” OR “mitigation” OR “mitigating” OR “rehabilitation” OR “protect” OR “protection” OR “protecting” OR “eliminate” OR “elimination” OR “eliminating” OR “restrict” OR “restrictions” OR “restricting” OR “resilience” OR “fight” OR “fighting” OR “confront” OR “confronting” OR “combat” OR “combating” OR “suppress” OR “suppression” OR “suppressing” OR “manage” OR “management” OR “managing” OR “organize” OR “organizing” OR “organising” OR “prepare” OR “preparation” OR “preparing” OR “preparedness” OR “against”.
