# Supplementary material for: Global Experiences of Community Responses to COVID-19: A Systematic Literature Review
Source: Front Public Health. 2022 Jul 19;10:907732. doi: 10.3389/fpubh.2022.907732 (PMC9343721; doi:10.3389/fpubh.2022.907732)
Supplement: Supplementary file 2 [file Data_Sheet_2.docx]

**Supplementary Material 2: Mixed Methods Appraisal Tool (MMAT), version 2018 (^[[1]](#endnote-1)^)**

**1. Qualitative**

Q1. Is the qualitative approach appropriate to answer the research question?

Q2. Are the qualitative data collection methods adequate to address the research question?

Q3. Are the findings adequately derived from the data?

Q4. Is the interpretation of results sufficiently substantiated by data?

Q5. Is there coherence between qualitative data sources, collection, analysis and interpretation?

**2. Quantitative randomized controlled trials**

Q1. Is randomization appropriately performed?

Q2. Are the groups comparable at baseline?

Q3. Are there complete outcome data?

Q4. Are outcome assessors blinded to the intervention provided?

Q5. Did the participants adhere to the assigned intervention?

**3. Quantitative non-randomized studies**

Q1. Are the participants representative of the target population?

Q2. Are measurements appropriate regarding both the outcome and intervention (or exposure)?

Q3. Are there complete outcome data?

Q4. Are the confounders accounted for in the design and analysis?

Q5. During the study period, is the intervention administered (or exposure occurred) as intended?

**4. Quantitative descriptive studies**

Q1. Is the sampling strategy relevant to address the research question?

Q2. Is the sample representative of the target population?

Q3. Are the measurements appropriate?

Q4. Is the risk of nonresponse bias low?

Q5. Is the statistical analysis appropriate to answer the research question?

**5. Mixed methods**

Q1. Is there an adequate rationale for using a mixed method design to address the research question?

Q2. Are the different components of the study effectively integrated to answer the research question?

Q3. Are the outputs of the integration of qualitative and quantitative components adequately interpreted?

Q4. Are divergences and inconsistencies between quantitative and qualitative results adequately addressed?

Q5. Do the different components of the study adhere to the quality criteria of each tradition of the methods involved?

**Quality appraisal of included studies (Mixed Methods Appraisal Tool scores)**

| Author, Year, [Ref number] | Study design | Q1 | Q2 | Q3 | Q4 | Q5 | Comments | MMAT score |
| --- | --- | --- | --- | --- | --- | --- | --- | --- |
| Ansari et al. (2021) [42] | Quantitative descriptive | -- | -- | √ | √ | √ | The sampling strategy was not clearly reported in this study. Thus, it is difficult to judge whether participants are representative of the target population. | 60% |
| Apata et al. (2021) [43] | Mix method | √ | √ | × | × | × | The integration of the qualitative and quantitative components was not adequately interpreted, and the qualitative and quantitative components of the study were lower than the quality criteria. | 40% |
| Aulandez et al. (2021) [44] | Qualitative | √ | × | -- | √ | × | The data collection process is implicit in the paper and is not explicitly stated. The thematic approach for data analysis is not clearly explained. | 40% |
| Bahagia et al. (2020) [45] | Qualitative | √ | √ | × | √ | × | The findings are adequately derived from the data. The interpretation of the results is somewhat superficial. | 60% |
| Baratta et al. (2021) [46] | Mix method | √ | √ | √ | -- | × | Mixed methods is an appropriate method to address the study question. However, the qualitative components of the study did not follow a strict data analysis process. | 60% |
| Biro-Hannah (2021) [47] | Qualitative | √ | √ | × | √ | × | The data collection process is implicit in the paper and is not explicitly stated. The quality of the qualitative data analysis is not high. | 60% |
| Cheng et al. (2020) [48] | Qualitative | √ | √ | √ | √ | × | The study followed a classical analytical process of a qualitative study. However, the links between data sources, collection, analysis, and interpretation are unclear. | 80% |
| Cheng et al. (2020) [49] | Quantitative non-randomized studies | -- | √ | × | × | √ | This study did not provide complete outcome data. Moreover, the confounders were not accounted for in the design and analysis. | 40% |
| Durmuş et al. (2020) [50] | Quantitative non-randomized studies | √ | √ | √ | × | √ | The study followed a classical analytical method of a quantitative study. However, the confounders were not accounted for in the design and analysis. | 80% |
| Frimpong et al. (2021) [51] | Qualitative | √ | √ | √ | √ | × | The study followed a classical analytical method of a quantitative study. However, the links between data sources, collection, analysis, and interpretation are unclear. | 80% |
| George et al. (2021) [52] | Quantitative | √ | √ | √ | √ | √ | The study followed a strict analytical process of a quantitative study. | 100% |
| Ha et al. (2020) [53] | Qualitative | √ | √ | √ | √ | √ | The study followed a strict analytical process of a qualitative study. | 100% |
| Hutchings et al. (2021) [54] | Qualitative | √ | √ | √ | √ | × | The study followed a classical analytical method of quantitative study. However, the links between data sources, collection, analysis, and interpretation are unclear. | 80% |
| Juhn et al. (2020) [55] | Quantitative descriptive | √ | √ | √ | -- | × | The nonresponse rate of this study is somewhat high. Moreover, the link between the study conclusion and statistical results is unclear. | 60% |
| Kwok et al. (2020) [56] | Quantitative non-randomized studies | -- | × | √ | √ | √ | The researchers of the present study cannot determine if the participants are representative of the target population since there is no clear description of the sampling method. Furthermore, the reliability and validity of the outcome variable measurement were not reported. | 60% |
| Lim et al. (2021) [57] | Qualitative | √ | √ | √ | √ | √ | The study followed a strict analytical process of a qualitative study, including participant selection, data collection, data analysis, and interpretation of the results. | 100% |
| McCalman et al. (2021) [58] | Qualitative | √ | √ | √ | √ | × | The study followed a strict analytical process of a qualitative study. However, it seems the study failed to have the analytic sharpness needed in qualitative content analysis. | 80% |
| McConachie et al. (2020) [59] | Mix method | √ | √ | × | × | × | The integration of the qualitative and quantitative components was not adequately interpreted, and the qualitative and quantitative components of the study were lower than the quality criteria. | 40% |
| Narasri et al. (2020) [60] | Qualitative | √ | × | √ | √ | √ | This is a qualitative study in compliance with standard analytical procedures. However, two focus group interviews seem insufficient to collect enough qualitative data for analysis, limiting the results. | 80% |
| Omboni et al. (2021) [61] | Quantitative non-randomized studies | -- | √ | √ | × | √ | It is unclear if the participants are representative of the target population, and the confounders were not accounted for in the design and analysis. | 60% |
| Patel et al. (2022) [62] | Quantitative descriptive | -- | -- | √ | √ | √ | The sampling strategy was not clearly reported in this study. Thus, it is difficult to judge if the participants are representative of the target population. | 60% |
| Peng et al. (2020) [63] | Quantitative non-randomized studies | √ | √ | √ | -- | √ | This is a qualitative study in compliance with standard analytical procedures. However, the researchers of the present study are not sure exhaustive confounders were accounted for in the design and analysis. | 80% |
| Pruitt et al. (2021) [64] | Quantitative descriptive | -- | -- | √ | √ | √ | The sampling strategy was not clearly reported in this study. Therefore, it is difficult to determine if the participants are representative of the target population. | 60% |
| Vanhamel et al. (2021) [65] | Qualitative | √ | √ | √ | √ | × | The study followed a strict analytical process of a qualitative study. However, it seems the study failed to have the analytic sharpness needed in qualitative content analysis. | 80% |
| Villani et al. (2020) [66] | Qualitative | √ | √ | -- | √ | × | The results lack richness and systematicness, making it hard for the reader to capture the experiences of the community-health partnership response in mitigating the challenges brought about by COVID-19. | 60% |
| Wallis et al. (2020) [67]  United Kingdom *** | Quantitative descriptive | × | -- | √ | √ | √ | The patient survey was distributed to the first 333 participants of the London North West University Healthcare NHS Trust (LNWUHT) community testing programme, which means the sampling strategy is not probability sampling. Accordingly, it is difficult to determine if the participants are representative of the target population. | 60% |
| Wong et al. (2021) [68]  Hong Kong *** | Mix method | √ | √ | √ | -- | × | Mixed methods is an appropriate method to address the study question. However, the qualitative components of the study did not follow a strict data analysis process. | 60% |
| Zhang et al. (2020) [69]  China *** | Qualitative | √ | -- | √ | × | √ | The data collection methods of this study seem unable to acquire enough qualitative data that reaches theoretical saturation. Additionally, the quotes provided to justify the themes are not adequate. | 60% |
| Zhu et al. (2020) [70]  China *** | Quantitative non-randomized studies | × | √ | √ | -- | √ | It seems that the participants (patients in trauma center of two hospitals) do not represent the target population (city-wide patients in trauma centers). Furthermore, the researchers of the present study are unsure if the exhaustive confounders were accounted for in the design and analysis. | 60% |

*Q1, Q2, Q3, Q4, Q5 refers to the question number of the scales. Studies of different design types was appraised with different scales shown above. √refers to “Yes”, × refers to “No”, -- refers to “Can’t tell”.*

1. . Pluye P, Robert E, Cargo M, Bartlett G, O’Cathain A, Griffiths F, et al. Proposal: A Mixed Methods Appraisal Tool for Systematic Mixed Studies Reviews. Available online at: http://mixedmethodsappraisaltoolpublic.pbworks.com/w/page/24607821/FrontPage (accessed January 14, 2022). [↑](#endnote-ref-1)
